# Altered respiratory microbiota composition and functionality associated with asthma early in life

**DOI:** 10.1186/s12879-020-05427-3

**Published:** 2020-09-22

**Authors:** Mohammad T. AL Bataineh, Rifat A. Hamoudi, Nihar R. Dash, Rakhee K. Ramakrishnan, Mohamad A. Almasalmeh, Hanan A. Sharif, Mohamed S. Al-Hajjaj, Qutayba Hamid

**Affiliations:** 1 Clinical Sciences Department, College of Medicine, University of Sharjah, Post Code: 27272, Sharjah, United Arab Emirates; 2 Sharjah Institute for Medical Research, University of Sharjah, Sharjah, United Arab Emirates; 3 Division of Surgery and Interventional Science, University College London, London, UK; 4 University Hospital Sharjah, Sharjah, United Arab Emirates; 5 Meakins-Christie Laboratories, McGill University, Montreal, QC Canada

**Keywords:** Asthma, Microbiota, Fungal

## Abstract

**Background:**

The microbiota of the respiratory tract has an important role in maintaining respiratory health. However, little is known on the respiratory microbiota in asthmatic patients among Middle Eastern populations. This study investigated the respiratory microbiota composition and functionality associated with asthma in Emirati subjects.

**Methods:**

We performed 16S rRNA and ITS2-gene based microbial profiling of 40 expectorated sputum samples from adult and pediatric Emirati individuals averaging 52 and 7 years of age, respectively with or without asthma.

**Results:**

We report bacterial difference belonging to *Bacteroidetes*, *Firmicutes*, *Fusobacteria* and *Proteobacteria* phyla between asthmatic and non-asthmatic controls. Similarly, fungal difference belonging to *Ascomycota*, *Basidiomycota* phyla and other unclassified fungi. Differential abundance testing among asthmatic individuals with relation to Asthma Control Test show a significant depletion of *Penicillium aethiopicum* and *Alternaria* spp., among poorly controlled asthmatics. Moreover, data suggest a significant expansion of *Malassezia* spp. and other unclassified fungi in the airways of those receiving steroids and leukotriene receptor antagonists’ combination therapy, in contrast to those receiving steroids alone. Functional profiling from 16S data showed marked differences between pediatric asthmatic and non-asthmatic controls, with pediatric asthmatic patients showing an increase in amino acid (*p*-value < 5.03 × 10^− 7^), carbohydrate (*p*-value < 4.76 × 10^− 7^), and fatty acid degradation (*p*-value < 6.65 × 10^− 7^) pathways, whereas non-asthmatic controls are associated with increase in amino acid (*p*-value < 8.34 × 10^− 7^), carbohydrate (*p*-value < 3.65 × 10^− 7^), and fatty acid (*p*-value < 2.18 × 10^− 6^) biosynthesis pathways in concordance with enterotype composition.

**Conclusions:**

These differences provide an insight into respiratory microbiota composition in Emirati population and its possible role in the development of asthma early in life. This study provides important information that may eventually lead to the development of screening biomarkers to predict early asthma development and novel therapeutic approaches.

## Background

Specialized microbial communities composed of bacteria, viruses and fungi termed as “respiratory microbiota” inhabit the human respiratory tract spanning from nostrils to the alveoli. The respiratory microbiota is highly dynamic, constantly evolving and influenced by multiple factors including host and environment [1, 2]. The respiratory microbiota is crucial for the maintenance of respiratory physiology and homeostasis [3]. It plays a significant role in the maturation and maintenance of respiratory immune responses and provides resistance to respiratory pathogen colonization [4]. At the same time, it has also been implicated in the structural development and morphogenesis of the respiratory tract [5, 6] as well as the development of mucosal immunity [7, 8].

The composition of the respiratory microbiota is increasingly being characterized in humans. Bacterial families *Dolosigranulum* spp.*, Corynebacterium* spp. [9], viral families *Anelloviridae* [10, 11], and fungi such as *Aspergillus* spp.*, Penicillium* spp.*, Candida* spp.*, Alternaria* spp. [12] populate the upper respiratory tract. Similarly, the lower respiratory tract is dominantly colonized by bacteria including *Haemophilus* spp.*, Moraxella* spp.*, Streptococcus* spp., *Staphylococcus* spp.*, Firmicutes* and *Bacteroidetes,* and fungal families such as *Eremothecium, Systenostrema* and *Malassezia* [13–15]. Characterization of the microbial communities residing in spatial niches along the respiratory tract is essential to elucidate the complex roles played by the respiratory microbiota in the pathogenesis of respiratory diseases.

Recent advances in our understanding of the respiratory microbiota composition and its alteration in diversity or abundance called “dysbiosis” has been linked to several chronic respiratory diseases such as asthma, cystic fibrosis, bronchiectasis, and chronic obstructive pulmonary disease [4, 16]. Asthma is a healthcare priority with significant social and economic impact on societies. Studies have linked nasopharyngeal colonization with *Streptococcus* spp., *Moraxella* spp., *Haemophilus* spp. *Prevotella* spp., and respiratory syncytial virus, early in life to the development of lower respiratory tract infections, consecutive atopic disease and future asthma [17–19]. In particular, early asymptomatic *Streptococcus* colonization strongly correlated with subsequent wheezing and asthma risk. An apparent disturbance in the characteristic composition of bacterial communities was observed in asthmatic airways when compared to their healthy counterparts [20, 21]. The bronchial airway microbiota composition and diversity significantly correlated with the degree of bronchial hyperresponsiveness in suboptimally controlled asthmatics [22]. Higher abundance of *Proteobacteria* is frequently observed in asthma patients [20, 22]. Bronchial microbiota also shows variations across the different endotypes of asthma [23]. Furthermore, the airway expansion of specific genera of gram-negative bacteria was noticed to induce corticosteroid resistance in asthmatic patients, indicating that the composition of airway microbiota may influence corticosteroid responsiveness in asthma [24].

The precise understanding of the composition of the respiratory microbiota, the mechanisms by which these microbes interact with host immunity, and their functional effects on the pathogenesis, exacerbations, and comorbidities of chronic respiratory diseases such as asthma is still unclear and need factual elucidation. Further, we are still unsure how the structural ligands and metabolites from these microbes interact with the host and alter the development and progression of respiratory diseases. Here, we investigated the composition, diversity and functionality of respiratory microbiome in a cohort of pediatric and adult asthmatic patients using sputum samples. We further, characterized the alterations in respiratory microbiota with age especially among the asthmatic population.

## Methods

Hospital Ethics and Research Committee, a local research ethics committee at the University Hospital Sharjah, UAE approved the study protocol (REC number: UHS-HERC- 039 -09042018). All subjects participating in the study supplied informed consent.

In this case control study, we collected 40 spontaneous expectorated sputum samples from Emirati citizens. Spontaneous coughed up sputum (expectorated phlegm/mucous) was the first preference of sample collection whenever possible in all subjects. The subjects were provided with a labelled sputum container. They were asked to take a deep breath, hold for a few seconds, exhale, repeat two or three times, and then cough: sputum was collected after a productive cough. Sputum induction was sometimes used in subjects especially in children when sputum could not be expectorated spontaneously. Sputum induction was performed under close medical supervision with nebulization and nasopharyngeal suction. Expectorated sputum was collected in a sterile container stored immediately into liquid nitrogen and then transferred to − 80 °C for further analysis. DNA extraction, PCR, Sequencing and Sequence processing were analyzed as described in supplementary document.

Information on the characteristics of the subjects in this study such as age, gender, body mass index (BMI), ethnicity, and animal exposure among others has been provided (Table 1).

**Table 1:** Demographics and Clinical Characteristics of Study Cohort

Characteristics	Adult Asthmatic (***n*** = 10)	Adult Healthy (***n*** = 10)	Pediatric Asthmatic (***n*** = 11)	Pediatric Healthy (***n*** = 9)
Age, years Mean (SD, range)	63.9 (12.7, 39)	40 (10.6, 32)	6.7 (4.1, 12)	8 (3.1, 8)
Ethnicity, ^a^Emirati (%)	100%	100%	100%	100%
Gender (M%, F %)	30:70	20:80	45:55	56:44
BMI (Kg/m^2^) Mean (SD, range)	31.5 (6.7, 21)	25.3 (4.7, 17)	21.6 (7.2, 20.7)	18.9 (5.2, 13.9)
Animal exposure (yes %)	0%	20%	9%	11%
Asthma Control Test Mean (SD, range)	18 (3.1, 10)	N/A	17.9 (4.4, 15)	N/A

N/A Not Applicable

^a^Native UAE citizens

We have also assessed asthma symptoms by collecting a patient-completed Asthma Control Test (ACT) questionnaire as previously described, scoring < 16 as uncontrolled, 16–19 as partially controlled, and 20–25 as controlled [25]. In summary, participants were residents of Sharjah, UAE. Asthma patients were defined as those individuals who had a current diagnosis of asthma, for example, by being on the outpatient asthma clinics registrar. Most of the asthma patients were on inhaled corticosteroids and scoring on average 18 per ACT. Controls were defined as individuals who on questioning did not report having current or previous asthma, eczema or hay fever. Use of antibiotics and/or prescribed probiotics in the past 3 months, any form of smoking, other respiratory diseases or infections among the participants were the exclusion criteria used in this study (Table 1).

### Functional profiling from 16S data

Gene family abundances from Kegg Orthology (KO) functional space were computed from 16S OTU data and GreenGenes taxonomic annotations with Phylogenetic Investigation of Communities by Reconstruction of Unobserved States 2 (PICRUSt2) [26]. Metabolic Modules were quantified from the PICRUSt KO abundance matrix with *GOmixer* R package [27]. Unsupervised Hierarchical Clustering analysis was carried out using pheatmap (https://cran.rproject.org/web/packages/pheatmap/pheatmap.pdf) function and subtree analysis was carried out using cutree (https://stat.ethz.ch/R-manual/Rpatched/library/stats/html/cutree.html) functions using in-house R script provided in supplementary document. Briefly, the cutree function allows the separation of the main tree into subtrees components using statistical methods [28]. The bar plots represent the relative difference between the average pathway abundance of pediatric asthmatics and healthy subjects of each pathway displaying ANOVA with *p*-value < 0.05. This was carried out using in-house R script.

### Statistical analysis

Alpha and beta diversity was calculated using Shannon and Bray-Curtis indices with significance of diversity differences was tested with an ANOVA. We excluded OTUs occurring with a count of less than 3 in at least 10% of the samples. Difference in bacterial microbiota composition was evaluated using PERMANOVA with smoking group as the main fixed factor. All statistical testing were performed in the R software environment.

## Results

### Sequence curation and metrics

Deep amplicon sequencing, combined with the principles of statistical ecology can be used to survey microbiome communities. The advent of massively parallel sequencing and increased computational power has enabled scientists to leverage big genomic data to answer biological questions. Here, we collected spontaneous expectorated sputum samples from 40 individuals in order to assess their microbiota composition. We sequenced 16Sv4 amplicons generated from DNA samples on a MiSeq and the resulting dataset had 5798 OTUs. An average of 42,657 quality-filtered reads were generated per sample. As demonstrated in the analytical flowchart (Fig. S1), sequencing quality for R1 and R2 was determined using FastQC 0.11.5, and visualized (Fig. S2). Similarly, we also sequenced ITS2 amplicons generated from DNA samples on the MiSeq. The resulting dataset had 4024 OTUs. An average of 41,635 quality-filtered reads were generated per sample. Sequencing quality for R1 and R2 was determined using FastQC 0.11.5, and visualized (Fig. S3). Next, we evaluated the taxonomic composition generated from these high-quality reads and classified them using Greengenes (v. 13_8) as the reference database for bacteria, and UNITE (v. 7.1) as the reference database for fungi. We aggregated OTUs into each taxonomic rank, and plotted the relative abundance of the most abundant ones. In the figure legends, the unfilled portion of the bar represents unclassified and lower-abundance taxa (Fig. S4).

### Respiratory microbiota of asthmatic subjects show lower levels of richness and complexity compared to healthy subjects

**Fig. 1:**
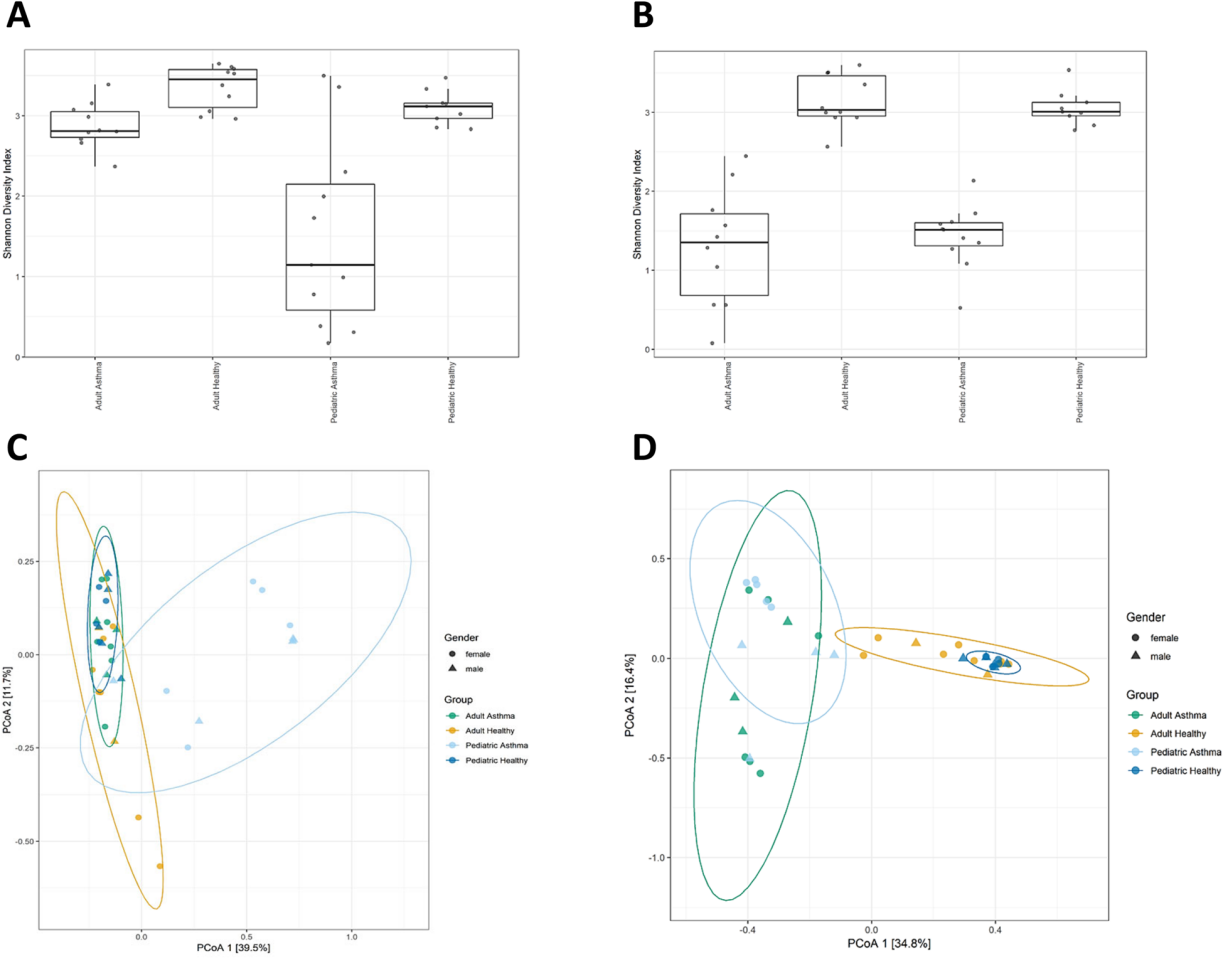
Lung microbiota of asthmatic subjects show lower levels of richness and complexity compared to healthy subjects. Evaluation of the alpha- and beta- diversity in the 40 analyzed samples. Panel showing alpha-diversity (Shannon index) computed and illustrated for each sample. ANOVA test determined significant differences in the Shannon diversity index based on different groups for bacterial data at *p* < 0.002 (**a**) and fungal data at *p* < 0.000 (**b**). To obtain a graphical representation of microbiome composition similarity among samples and beta-diversity, we summarized OTU abundances into Bray-Curtis dissimilarities and performed a PCoA ordination. Permutational analysis of variance (adonis R function, or Permanova) determined significant differences in beta-diversity among groups for bacterial data at *p* < 0.0009 (**c**) and fungal data at *p* < 0.0001 (**d**)

To assess richness of the 40 samples along with the evenness of bacterial and fungal populations for different asthmatic and healthy groups, we calculated alpha diversity using Shannon index. Next, to evaluate microbiome composition similarity across samples, we used abundance-weighted sample pair-wise differences using the Bray-Curtis dissimilarity. We observed a significant difference of bacterial and fungal populations among asthmatic groups compared to healthy groups (Fig. 1). Further, we conducted a post-hoc pairwise test of different groups (asthmatic and healthy) and noted a uniformity in the expectorated sputum fungal composition between Adult Asthma vs. Pediatric Asthma groups (p.adjusted > 0.129) (Table 2).

**Table 2:** Beta diversity post-hoc pairwise test of different groups

Pairs	R2	p.adjusted
16S (Bacterial) data		
Adult Asthma vs Pediatric Asthma	0.342	0.002
Adult Asthma vs Adult Healthy	0.118	0.023
Pediatric Asthma vs Pediatric Healthy	0.362	0.002
ITS2 (Fungal) data		
Adult Asthma vs Pediatric Asthma	0.097	**0.129**
Adult Asthma vs Adult Healthy	0.318	0.002
Pediatric Asthma vs Pediatric Healthy	0.427	0.002

### Relative abundances of most abundant bacterial and fungal taxa in asthmatics

**Fig. 2:**
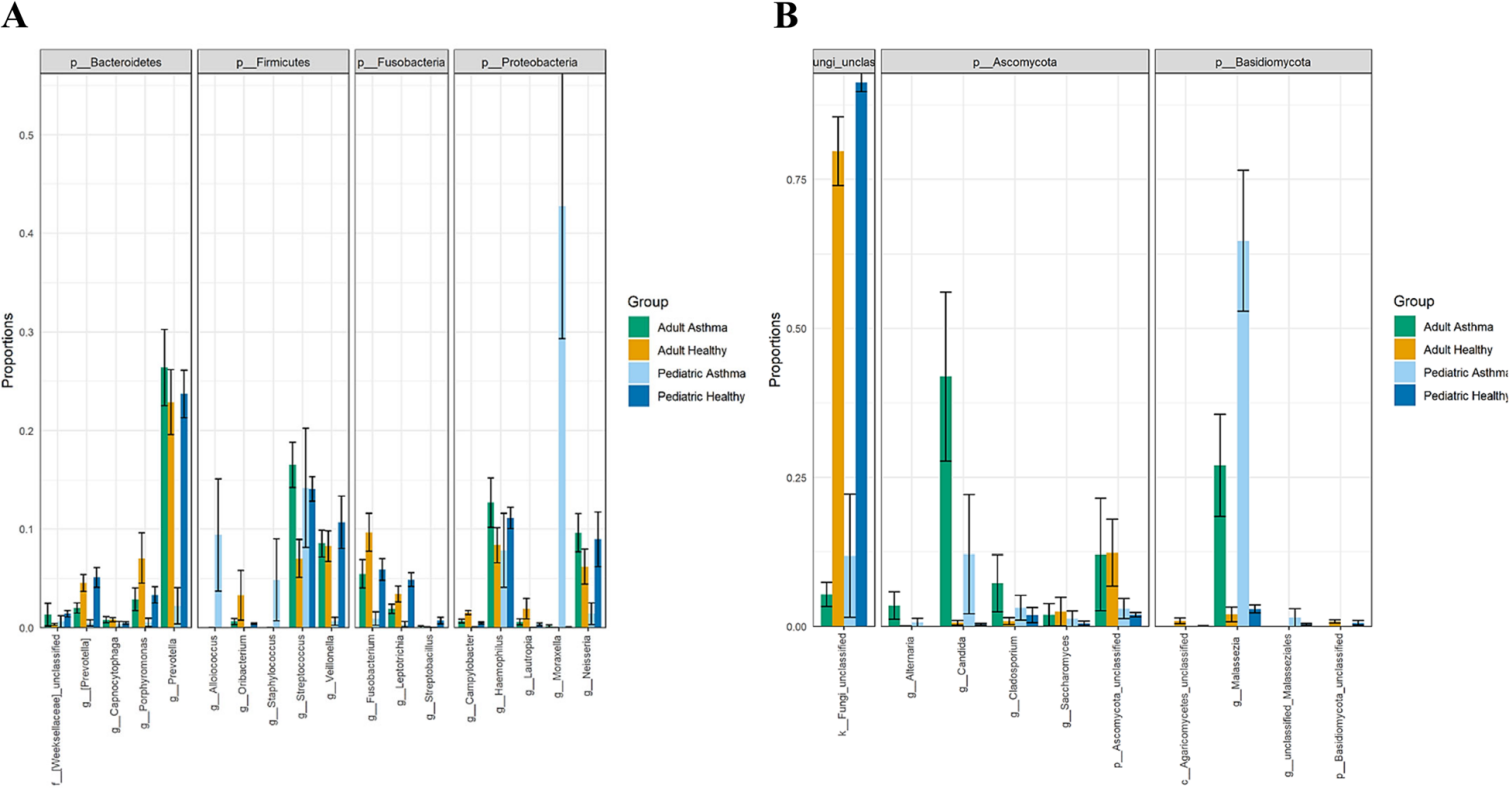
Relative abundances of the most abundant taxa*.* The following plot illustrates the mean and standard error of the relative abundances of the 5 most abundant genus-level taxa within the 4 most abundant Phyla for bacteria (**a**) and fungi (**b**). The Genus-level plots are grouped according to Phylum along the x-axis. The groupings along the y-axis represent the column of metadata. The barplot colors represent different groups

In order to identify the important respiratory microbiota members that significantly differ between asthmatic and healthy groups as suggested in Fig. 1, we evaluated relative abundances of the five most abundant genus-level taxa within the four most abundant Phyla for bacteria and fungi (Fig. 2). We noted a significant bacterial difference belonging to *Bacteroidetes*, F*irmicutes*, F*usobacteria* and P*roteobacteria* phyla. Pediatric asthma group showed abundance in *Streptococcus* spp., *Moraxella* spp. among others. Moreover, we determined depletion of unclassified fungi in asthmatic groups and irrespective of age (Fig. 2b), we also noted significant abundance of *Malassezia* spp. and C*andida* spp. among others in asthmatic groups. A more detailed table summarizing genera with significant differences in relative abundances of bacterial and fungal overall diversity and prevalence among healthy and asthmatic groups, Kruskal-Wallace *P*-values as well as Benjamin-Hochberg corrected values are also reported (Table S1).

### Bacterial and fungal differential abundance among asthmatic individuals with relation to current medications and asthma control test (ACT)

**Fig. 3:**
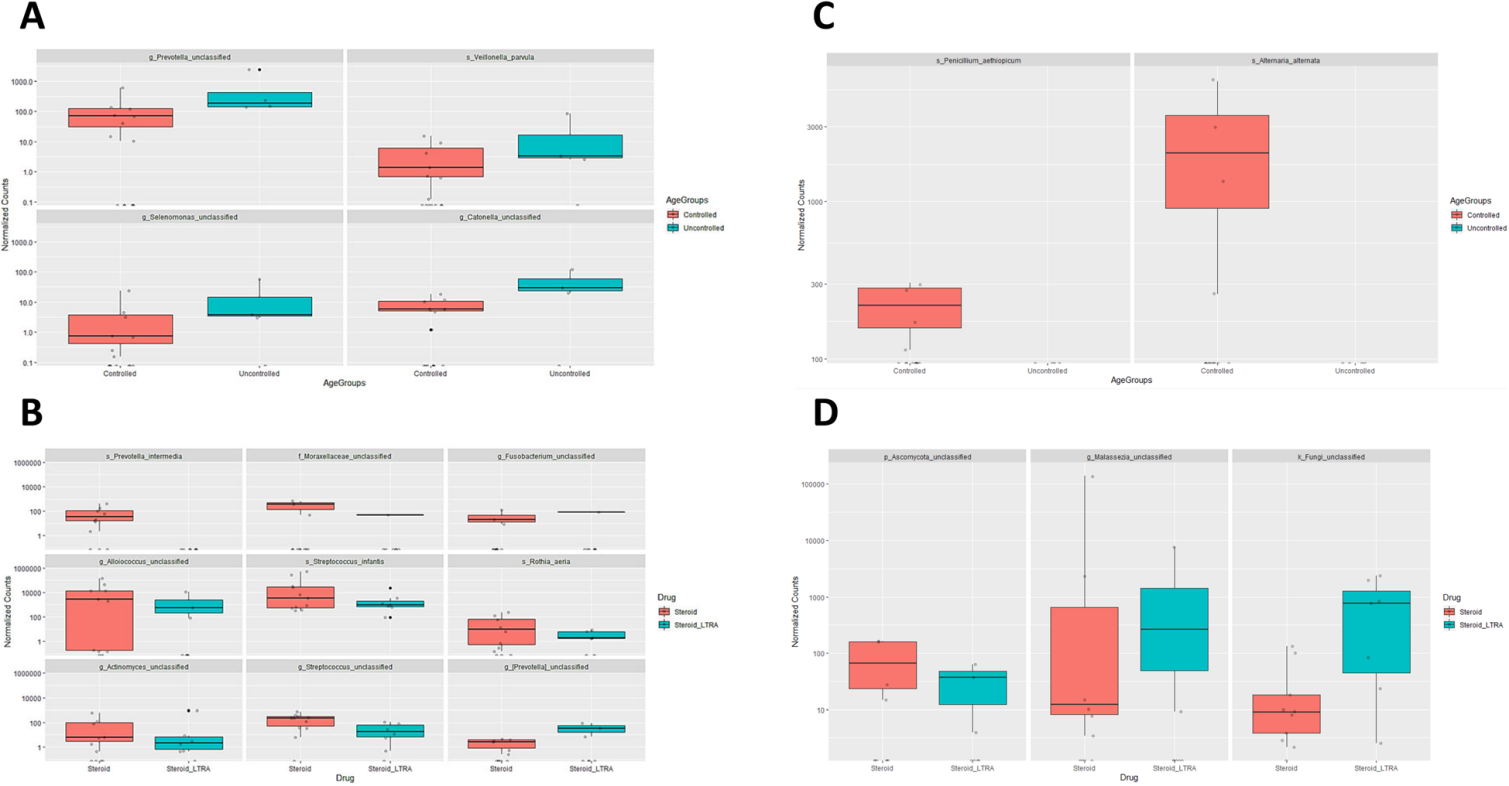
Investigation of bacterial and fungal differential abundance among asthmatic individuals with relation to current medications and asthma control test (ACT). Differential abundance testing using DESeq2, R package to identify differentially abundant taxa among drug + ACT variables. The bar plot reports bacterial genera **(a, b)** and fungal genera (**c, d**) with significant abundance with relation to drug and ACT scores at a *p*-value < 0.05 and the Log2Fc / Fold Change > 1.5 or < − 1.5 for both bacterial and fungal genera. Controlled and uncontrolled ACT scores as well as steroid and steroid + LTRA current treatment corresponding abundance are colored in red and blue, respectively

DESeq2 R package was used to identify differentially abundant taxa among medications used or ACT group variables. Differential abundance testing identified four bacterial OTUs, and two fungal OTUs that were differentially abundant with relation to ACT test result (partially controlled and controlled group vs. uncontrolled group) (Fig. 3a, c). ACT is a self-administered test to identify those with poorly controlled asthma [25, 29]. Accordingly, we show a significant depletion of *Penicillium aethiopicum* and *Alternaria* spp., with poorly controlled subjects (Fig. 3c). Also, we report differences of nine bacterial OTUs and three fungal OTUs when treated with steroids and leukotriene receptor antagonists (LTRA) in contrast to treatment with steroids alone (Fig. 3b, d). In particular, we reveal expansion of *Malassezia* spp. and other unclassified fungi (Fig. 3d) and unclassified genre belonging to *Fusobacteria* and *Prevotella* (Fig. 3b) in the airways of those receiving steroids and LTRA combination therapy.

### Functional profiling of asthmatic and healthy microbiota based on PICRUSt2 analyses of 16S data

**Fig. 4:**
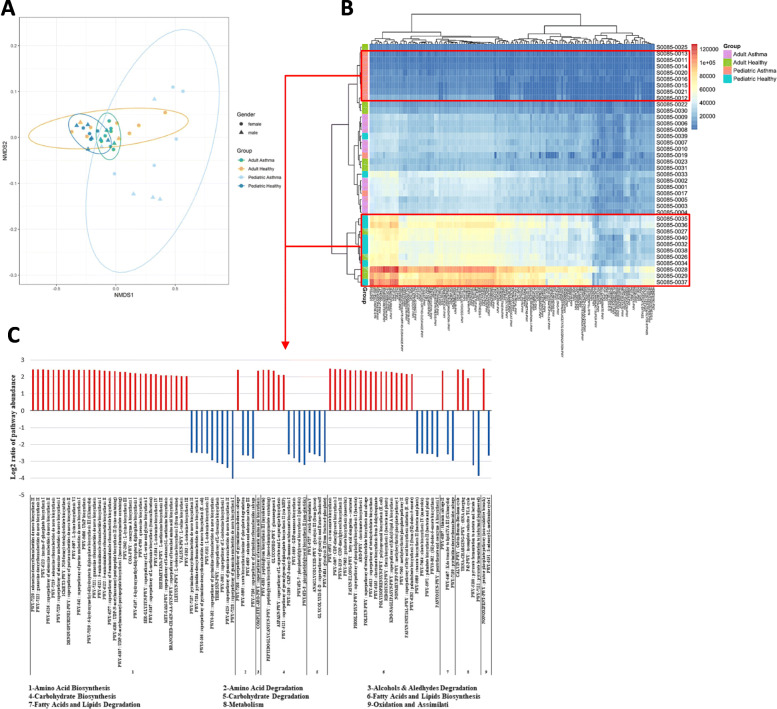
Functional profiling of asthmatic and healthy microbiomes based on PICRUSt2 analyses of 16S data. To obtain a graphical representation of PICRUSt2 functional composition among samples, we summarized PICRUSt abundances into Bray-Curtis dissimilarities and performed a NMDS ordination (**a**). Permutational analysis of variance (adonis R function, or Permanova) determined significant differences in beta-diversity among different groups at *p*-value < 0.0001. Significant functional pathways were plotted in a heatmap, only pediatric healthy and pediatric asthma groups from red boxes were used for B (**b**). The bar plot shows log2 ratios of the average pathway abundance for pediatric healthy and pediatric asthma subjects of each pathway displaying ANOVA with *p*-value < 0.05. Pediatric healthy and pediatric asthma more abundant pathways are colored in red and blue, respectively (**c**)

We used PICRUSt analysis to predict functional contribution of the bacterial microbiota in the samples from 16S OTU abundance data. Functional profiling showed significant overall differences in metabolic potential between asthmatics and healthy across age groups, especially among pediatric age groups (Fig. 4). Four hundred and ten significantly functional pathways were plotted in a heatmap showing a distinguished pattern of pathway enrichment in red or depletion in blue with relation to asthmatic and healthy groups (Fig. 4b). PICRUSt abundances permutational analysis of variance (adonis R function, or Permanova) determined significant differences in beta-diversity among different groups at *p*-value < 0.0001 (Fig. 4a). Moreover, derived from functional inference - PICRUSt2 data, average pathway abundance for pediatric asthmatic and healthy subjects of each pathway group was calculated using unsupervised hierarchical clustering followed by cutree to delineate the healthy versus asthmatic pediatric patients. Cutree was used to extract the pediatric asthmatic patients from the mixed tree based on differentially expressed pathways. The region delineated using the cutree was then used to calculate the relative difference between the average abundance in healthy and asthmatic pediatric subjects for each pathway showing significant change between the pediatric asthma and healthy individuals based on ANOVA *p* < 0.05 cutoff threshold for each pathway. We identified ninety pathways, in which pediatric asthmatic group show significant depletion in metabolic pathways implicated in amino acid, carbohydrate, and fatty acid biosynthesis, in contrast to significant enrichment of metabolic pathways involved in amino acid, carbohydrate, and fatty acid degradation in comparison to healthy pediatric group (Fig. 4c).

## Discussion

In this study, we explored the composition and functional contribution of respiratory microbiota in asthmatics and their possible role with relation to ACT scores and asthma medication in adult and pediatric age groups. First, we observed that respiratory microbiota of asthmatic subjects showed lower levels of richness and complexity compared to healthy subjects. Consistent with our findings, previous reports have suggested a strong effect for asthma on lung microbiota composition [30, 31]; however, very little has been reported on the role of fungal lung microbiota (mycobiota) in asthma. Here, we conducted a post-hoc pairwise test of different groups (asthmatic and healthy) and noted an intriguing uniformity in the expectorated sputum fungal composition between adult and pediatric asthma groups (p.adjusted > 0.129) (Table 2). This may suggest an important role for lung mycobiota in driving these compositional changes and their contribution to asthma pathogenesis in early life that persist with age. Indeed, colonization patterns in infancy seem to be a major determinant of respiratory disease later in life [4]. Perhaps fungal presence induces changes in the lung micro-environmental conditions that subsequently drives this compositional shift among bacterial communities to compensate. For example, enrichment of *Moraxella* species in asthmatic airways was found to interact negatively with multiple bacterial communities but positively with fungal communities suggesting complex interactions between the bacterial and fungal communities that may contribute to asthma pathogenesis [32] That said, it is important to note that limited fungal genome database may also explain the identified similarity between adult and pediatric groups. For example, recent check (July, 2020) of the NCBI Genome database https://www.ncbi.nlm.nih.gov/genome/browse#!/eukaryotes/fungi shown 6570 complete fungal genomes compared with > 252,000 complete bacterial genomes. Altogether, these observations are interesting to identify those microbial populations that are more abundant in asthma and to further understand their relevance to asthma pathogenesis and host immune interactions. Next, we identified significant relative abundances of *Bacteroidetes*, F*irmicutes*, *Fusobacteria* and *Proteobacteria* phyla in asthmatics (Fig. 2). Further and consistent with previous study, we noted significant abundance of *Malassezia* spp. and C*andida* spp. in asthmatic groups [15]. We also showed abundance in *Streptococcus* spp., *Moraxella* spp. among other fungi in Pediatric Asthma group, and consistent with a previous study that links nasopharyngeal colonization with these species during early life to the development of lower respiratory tract infections, consecutive atopic disease and future asthma [17]. Moreover, we unraveled for the first time a staggering depletion of unclassified fungi in asthmatic groups and irrespective of age (Fig. 2b). These fungi, as eukaryotes, probably contribute through unique metabolic pathways to microbial equilibrium and host interactions to protect against asthma development.

Third, differential abundance testing with relation to asthmatic medications used or ACT score variables unveiled a significant depletion of *Penicillium aethiopicum* and *Alternaria* spp., in poorly controlled subjects (low ACT scores) (Fig. 3c). Previous studies indicated that indoor exposure to *Penicillium* spp., and *Alternaria* spp., is associated with active asthma symptoms [33–35]. Further, the richness and composition of respiratory microbiota of asthmatic patients were found to be significantly altered by inhaled and oral corticosteroid use [22, 36, 37]. On the other hand, the airway microbiome can also influence the corticosteroid responsiveness in asthma. A study by Goleva et al., noted expansion of the pathogenic *Haemophilus parainfluenzae* in corticosteroid-resistant asthma, and stimulation of bronchoalveolar lavage (BAL) macrophages with *H. parainfluenzae* promoted the activation of MAPK pathway and subsequent inhibition of corticosteroid responses, in contrast to commensal *Prevotella melaninogenica* [24]. However, little is known about the leukotriene receptor antagonists (LTRA) correlation with microbiota in asthma. Here, we report for the first-time differences of nine bacterial OTUs and three fungal OTUs when treated with steroids and LTRA in contrast to treatment with steroids alone (Fig. 3b, d). Interestingly, we demonstrate expansion of *Malassezia* spp., unclassified fungi, and genre belonging to unclassified *Fusobacteria* and unclassified *Prevotella* in the airways of those receiving steroids and LTRA combination therapy. These data can be useful to provide screening biomarkers to predict responsiveness to asthma management.

Lastly, utilizing PICRUSt analysis of the bacterial microbiota we revealed significant overall differences in metabolic potential between asthmatics and healthy across age groups, especially among pediatric age groups (Fig. 4). These differences provide an insight into respiratory microbiota fluctuations in Emirati population and its possible role in asthma pathogenesis. Moreover, using unsupervised hierarchical clustering followed by cutree to delineate the healthy versus asthmatic pediatric patients we identified ninety pathways, in which pediatric asthmatic group show significant depletion in metabolic pathways implicated in amino acid, carbohydrate, and fatty acid biosynthesis, in contrast to significant enrichment of metabolic pathways involved in amino acid, carbohydrate, and fatty acid degradation in comparison to healthy pediatric group (Fig. 4c). Maintenance of metabolic homeostasis, such as regulation of glucose uptake, amino acid acquisition and lipid synthesis are tightly governed by catabolic and anabolic events that ultimately dictates cell growth and proliferation, especially early in life [38]. The compromised balance between the two, as observed in our study, may contribute to disease pathogenesis within the lung milieu. For instance, elevation in multiple catabolic biomarkers is an indicator of accelerated decline of muscle strength [39]. A recent report sheds light on the cross talk between airway microbiota and host airway cells in signaling anabolic and catabolic remodeling of the transplanted lung [40]. Therefore, the increased anabolic ability among asthmatic group may ultimately enhance airway remodeling and inflammation, hallmark features of asthma. For example, among these pathways, we observed significant depletion of inosine degradation (PWY-5695) (*p*-value < 3.15 × 10^− 7^), adenosine salvage (PWY-6609) (*p*-value < 5.48 × 10^− 7^), and pyrimidine salvage (PWY-7196) (*p*-value < 6.18 × 10^− 7^) (Fig. 4c) in pediatric asthmatics. Previous studies reports an important role for these purine metabolites in modulating lung inflammation [41, 42]. Here, we observed significant depletion of the gram-positive *Lactobacillus* among pediatric asthmatic groups (P. adj < 0.04) (Table S1). Interestingly, another study suggested that remodeling microbiota with *Lactobacillus* prolonged mice survival and reduced inflammation in the context of regulatory T cell dysfunction by restoring levels of inosine [43]. Moreover, other studies observed increased aerobic glycolysis and lactate production in asthma, which in turn promotes asthma development by T cell activation [44, 45]. Here however, we show a significant depletion of glycolysis pathways (ANAGLYCOLYSIS-PWY, GLYCOLYSIS-E-D, PWY-5484) (*p*-values < 3.13 × 10^− 7^, < 7.89 × 10^− 7^, < 1.96 × 10^− 7^ respectively) (Fig. 4c) in pediatric asthmatic group despite the reported increased relative abundance of lactic acid producing bacteria in this group, such as *Streptococcus* and *Enterococcus* [46] (P. adj < 0.04, < 0.05 respectively) (Table S1). Perhaps other major members of lung microbiota, such as *Moraxella* (% absolute 42.75%, P. adj < 0.00) (Table S1) in pediatric asthmatic group, in part explains the observed depletion of glycolysis pathways as previously reported to primarily drive gluconeogenesis [47]. Overall, these findings highlight the importance of respiratory microbiota in determining the airway microenvironment and thereby, influencing lung function.

Limitations of this study include that we used a small number of samples, which needs further validation on a larger cohort across populations with different environmental conditions and genetic background. We also used spontaneous expectorated sampling method that carry a high risk of cross-contamination of the lower respiratory tract microbiota with upper respiratory tract microbiota. Distinguishing between the two is difficult due to the anatomical link between both niches. That said, studies suggested that lung microbiota mostly resembles the upper respiratory tract microbiota [13, 48]. Fortunately, our expectorated sputum sampling is consistent with previous microbiome studies of bronchial sampling with high proportion of bacteria belonging to *Bacteroidetes*, *Firmicutes*, *Fusobacteria* and *Proteobacteria* phyla [21, 49]. Further, similar to our observation in pediatric asthmatics, a study noted that bacterial colonization of neonatal airways at 1 month of age was observed to predominate with pathogenic *Streptococcus pneumoniae*, *Haemophilus influenzae* or *Moraxella catarrhalis*, which in turn increased their risk and severity of wheeze as well as increased the total IgE and blood eosinophil counts by the age of 5 years, leading to their diagnosis of clinical asthma by age 5 and also implying their role in the development and progression of asthma [49]. Whereas another study by Huang et al., noted relative abundance of the airway microbiota belonging to other phylotypes, including members of *Comamonadaceae, Sphingomonadaceae, Oxalobacteraceae* bacterial families, correlated with the degree of bronchial hyperresponsiveness in patients with sub-optimally controlled asthma [22].

In conclusion, we identified respiratory bacterial and fungal species associated with asthmatic patients and shed more light on the functional impact of respiratory microbiota on asthma pathogenesis in correlation with management indices such as ACT test. Host and environmental factors dictates the respiratory microbiota makeup, which in turn will further shift the balance toward more inflammatory or protective role. These inflammatory responses govern the interplay between respiratory colonization and asthma development in a bidirectional manner that can be further complicated by asthma medications such as corticosteroids [4, 22, 24, 37]. Therefore, it is difficult to predict the potential contribution of the specific members of the airway microbiota towards asthma pathogenesis, underscoring the need for more functional studies and uniform sampling processes. We believe results from this study will further enhance our understanding of the composition and functionality of respiratory microbiota in asthmatic patients that can influence the potential manipulation of the microbiome as a therapeutic strategy for chronic respiratory diseases such as asthma. We hope that future work will provide a platform for better understanding of asthma pathophysiology. Most likely, these data can be useful to provide screening biomarkers to predict early asthma development and better management.

## Supplementary information

**Additional file 1:** Supplemental Materials and Methods.

**Additional file 2: Figure. S1**. Analytical flowchart outlining data curation and metrics used for analysis. **Figure. S2**. Bacterial sequence curation and analysis. We sequenced 16Sv4 amplicons generated from DNA samples on a MiSeq. MiSeq-generated Fastq files were quality-filtered and clustered into 97% similarity operational taxonomic units (OTUs) using the mothur software package [http://www.mothur.org]. The per-base raw Q30 (Phred33) scores of the forward sequencing read are summarized (A). Sequencing quality for R1 and R2 was determined using FastQC 0.11.5, the per-sequence averaged raw Q30 (Phred33) scores of the forward sequencing read are summarized (B). **Figure. S3**. Fungal sequence curation and analysis. We sequenced ITS2 amplicons generated from DNA samples on a MiSeq. MiSeq-generated Fastq files were quality-filtered and clustered into 97% similarity operational taxonomic units (OTUs) using the mothur software package [http://www.mothur.org]. The per-base raw Q30 (Phred33) scores of the forward sequencing read are summarized (A). Sequencing quality for R1 and R2 was determined using FastQC 0.11.5, the per-sequence averaged raw Q30 (Phred33) scores of the forward sequencing read are summarized (B). **Figure. S4**. Aggregated taxonomic composition. High quality reads were classified using Greengenes v. 13_8 as the reference database for bactria and UNITE (v. 7.1) as the reference database for fungi. We aggregated OTUs into each taxonomic rank, the aggregated taxa were visualized at each taxanomic rank using taxanomic bar plots and plotted the relative abundance of the most abundant ones for bacteria (A) and Fungi (B). The unfilled portion of the bar plots represent lower-abundance taxa.

**Additional file 3: Table S1**. Summary of Bacterial and Fungal diversity and Prevalence Among Healthy and Asthmatic Groups Using Kruskal-Wallace *P*-values and Benjamin-Hochberg Corrected Values.

## Data Availability

The data that support the findings of this study are available on request from the corresponding author. The data are not publicly available due to privacy or ethical restrictions.

## Abbreviations

ACT: Asthma Control Test
ANOVA: Analysis of variance
BMI: Body mass index
KO: Kegg Orthology
OUT: Operational Taxonomic Unit
PICRUSt2: Phylogenetic Investigation of Communities by Reconstruction of Unobserved States 2
PERMANOVA: Permutational analysis of variance
