# Supplementary material for: Altered respiratory microbiota composition and functionality associated with asthma early in life
Source: BMC Infect Dis. 2020 Sep 22;20:697. doi: 10.1186/s12879-020-05427-3 (PMC7510324; doi:10.1186/s12879-020-05427-3)
Supplement: Supplementary file 1 — Additional file 1: Supplemental Materials and Methods. [file 12879_2020_5427_MOESM1_ESM.docx]

**Supplemental Materials and Methods**

**DNA extraction, PCR, Sequencing and Sequence processing**

Specimens were placed into a MoBio PowerMag Soil DNA Isolation Bead Plate. DNA was extracted following MoBio’s instructions on a KingFisher robot. Bacterial 16S rRNA genes were PCR-amplified with dual-barcoded primers targeting the V4 region (515F 5’-GTGCCAGCMGCCGCGGTAA-3’, and 806R 5’-GGACTACHVGGGTWTCTAAT-3’), as per the protocol of Kozich et al. (2013)([1](#_ENREF_1)). Fungal ITS2 region were sequenced on an Illumina MiSeq (v. 3 chemistry) using the dual barcoding protocol of Kozich et al. (2013)([1](#_ENREF_1)). Primers (ITSF 5’-CCTCCGCTTATTGATATGC-3’, ITSR 5’-CCGTGARTCATCGAATCTTTG-3’) and PCR conditions used for ITS2 sequencing were described by Gweon et al. (2015)([2](#_ENREF_2)). Next, amplicons were sequenced with an Illumina MiSeq using the 300-bp paired-end kit (v.3). Sequences were denoised, taxonomically classified using Greengenes (v. 13_8) as the reference database, and clustered into 97%-similarity operational taxonomic units (OTUs) with the mothur software package (v. 1.39.5) (Schloss et al. 2009)([2](#_ENREF_2)), following the recommended procedure (<https://www.mothur.org/wiki/MiSeq_SOP>; accessed Nov 2017). Paired-end reads were merged and curated to reduce sequencing error as previously described (Huse et al. 2010)([3](#_ENREF_3)). The fungal processing pipeline was identical as the one used for bacteria, except for the following differences: (1) paired-end reads were trimmed at the non-overlapping ends, and (2) high quality reads were classified using UNITE (v. 7.1) (Kõljalg et al. 2005)([4](#_ENREF_4)) as the reference database.

**Quality Control**

The possibility for contamination was examined by co-sequencing DNA amplified from expectorated sputum specimens and from four each of template-free controls and extraction kit reagents treated the same way as the specimens. Two positive controls, consisting of cloned SUP05 DNA, were also added (number of copies = 2*10^6). Operational taxonomic units were considered putative contaminants (and were removed) if their mean abundance in controls reached or surpassed 25% of their mean abundance in specimens.

**References:**

1. Kozich JJ, Westcott SL, Baxter NT, Highlander SK, Schloss PD. Development of a dual-index sequencing strategy and curation pipeline for analyzing amplicon sequence data on the miseq illumina sequencing platform. *Applied and environmental microbiology* 2013;79:5112-5120.

2. Gweon HS, Oliver A, Taylor J, Booth T, Gibbs M, Read DS, Griffiths RI, Schonrogge K. Pipits: An automated pipeline for analyses of fungal internal transcribed spacer sequences from the illumina sequencing platform. *Methods in ecology and evolution* 2015;6:973-980.

3. Huse SM, Welch DM, Morrison HG, Sogin ML. Ironing out the wrinkles in the rare biosphere through improved otu clustering. *Environmental microbiology* 2010;12:1889-1898.

4. Koljalg U, Larsson KH, Abarenkov K, Nilsson RH, Alexander IJ, Eberhardt U, Erland S, Hoiland K, Kjoller R, Larsson E, Pennanen T, Sen R, Taylor AF, Tedersoo L, Vralstad T, Ursing BM. Unite: A database providing web-based methods for the molecular identification of ectomycorrhizal fungi. *The New phytologist* 2005;166:1063-1068.

Supplementary code. Detailed information about Unsupervised Hierarchical Clustering analysis using pheatmap function and subtree analysis using cutree

**# Unsupervised hierarchical clustering**

library(pheatmap)

library(RColorBrewer)

library(gplots)

setwd("D:/AProf/SL stuff/Sharjah/Projects/Bataineh/Asthma/16s/Figure for paper/Feb 2020")

dd = read.table("Trasnposed_path_abun_subcluster.txt", header=TRUE, row.names = 1, stringsAsFactors = FALSE)

metadata = read.table("metadata.txt", header=TRUE, row.names = 1, stringsAsFactors = FALSE)

out <- pheatmap(dd, angle_col=45, annotation_row = metadata, cex = 0.5)

rownames(dd[out$tree_row[["order"]],]) # reorder

colnames(dd[,out$tree_col[["order"]]])

write.table(data.frame(gene = rownames(dd)[out$rowInd]), 'out.csv', row.names = TRUE, quote = FALSE, sep = ',')

**# Calculating abundance using ANOVA**

setwd("D:/AProf/SL stuff/Sharjah/Projects/Bataineh/Asthma/16s/Figure for paper/Feb 2020")

tabx <- read.table("Trasnposed_path_abun_subcluster - ANOVA.txt", header=TRUE, sep="\t", quote="", comment.char="", na.strings="#N/A")

asthmadata <- tabx[,-c(1:4)]

head(asthmadata)

diagnosis <- c(tabx[1:20,4])

**# Calculating the log2**

totalgrp <- dim(asthmadata)[2]

grp1 <- as.numeric(table(diagnosis)[1])

grp2start <- grp1 + 1

grp2 <- totalgrp - grp1

**# Setup log2 in normal space**

asthma <- asthmadata

log2mat = matrix(nrow=nrow(asthma), ncol=1)

for (i in 1:nrow(asthmadata))

{

rec <- as.numeric(asthmadata[i,])

log2mat[i] <- log2(mean(rec[1:grp1])/mean(rec[grp2start:totalgrp]))

}

**# Setup ANOVA**

SigProb <- function(y, x)

{

oneway.test(y ~ x, var.equal=TRUE)$p.value

}

asthma <- asthmadata

index = matrix(nrow=nrow(asthma), ncol=1)

pvals = matrix(nrow=nrow(asthma), ncol=1)

for (i in 1:nrow(asthmadata))

{

rec <- as.numeric(asthmadata[i,])

index[i] <- (SigProb(rec, diagnosis) < 0.05)

pvals[i] <- SigProb(rec, diagnosis)

}

table(index)

x <- asthma

x <- cbind(tabx, log2mat, pvals)

x <- x[index,]

write.table(x, file="ANOVA_probes.csv", sep=",", row.names=TRUE)
