# Supplementary material for: Altered respiratory microbiota composition and functionality associated with asthma early in life
Source: BMC Infect Dis. 2020 Sep 22;20:697. doi: 10.1186/s12879-020-05427-3 (PMC7510324; doi:10.1186/s12879-020-05427-3)
Supplement: Supplementary file 2 — Additional file 2: Figure. S1. Analytical flowchart outlining data curation and metrics used for analysis. Figure. S2. Bacterial sequence curation and analysis. We sequenced 16Sv4 amplicons generated from DNA samples on a MiSeq. MiSeq-generated Fastq files were quality-filtered and clustered into 97% similarity operational taxonomic units (OTUs) using the mothur software package [http://www.mothur.org]. The per-base raw Q30 (Phred33) scores of the forward sequencing read are summarized (A). Sequencing quality for R1 and R2 was determined using FastQC 0.11.5, the per-sequence averaged raw Q30 (Phred33) scores of the forward sequencing read are summarized (B). Figure. S3. Fungal sequence curation and analysis. We sequenced ITS2 amplicons generated from DNA samples on a MiSeq. MiSeq-generated Fastq files were quality-filtered and clustered into 97% similarity operational taxonomic units (OTUs) using the mothur software package [http://www.mothur.org]. The per-base raw Q30 (Phred33) scores of the forward sequencing read are summarized (A). Sequencing quality for R1 and R2 was determined using FastQC 0.11.5, the per-sequence averaged raw Q30 (Phred33) scores of the forward sequencing read are summarized (B). Figure. S4. Aggregated taxonomic composition. High quality reads were classified using Greengenes v. 13_8 as the reference database for bactria and UNITE (v. 7.1) as the reference database for fungi. We aggregated OTUs into each taxonomic rank, the aggregated taxa were visualized at each taxanomic rank using taxanomic bar plots and plotted the relative abundance of the most abundant ones for bacteria (A) and Fungi (B). The unfilled portion of the bar plots represent lower-abundance taxa. [file 12879_2020_5427_MOESM2_ESM.pdf]

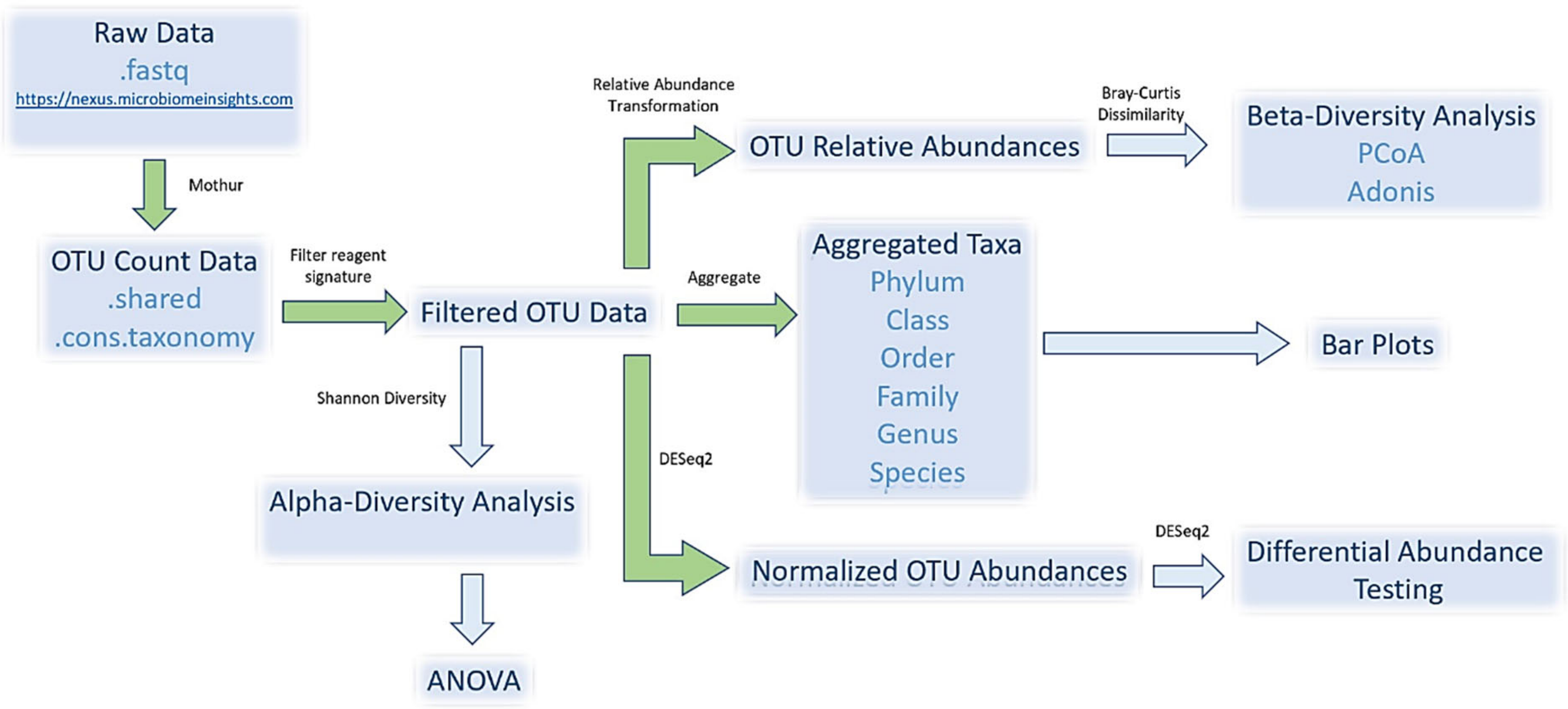

Fig. S1. Analytical flowchart outlining data curation and metrics used for analysis.

A B

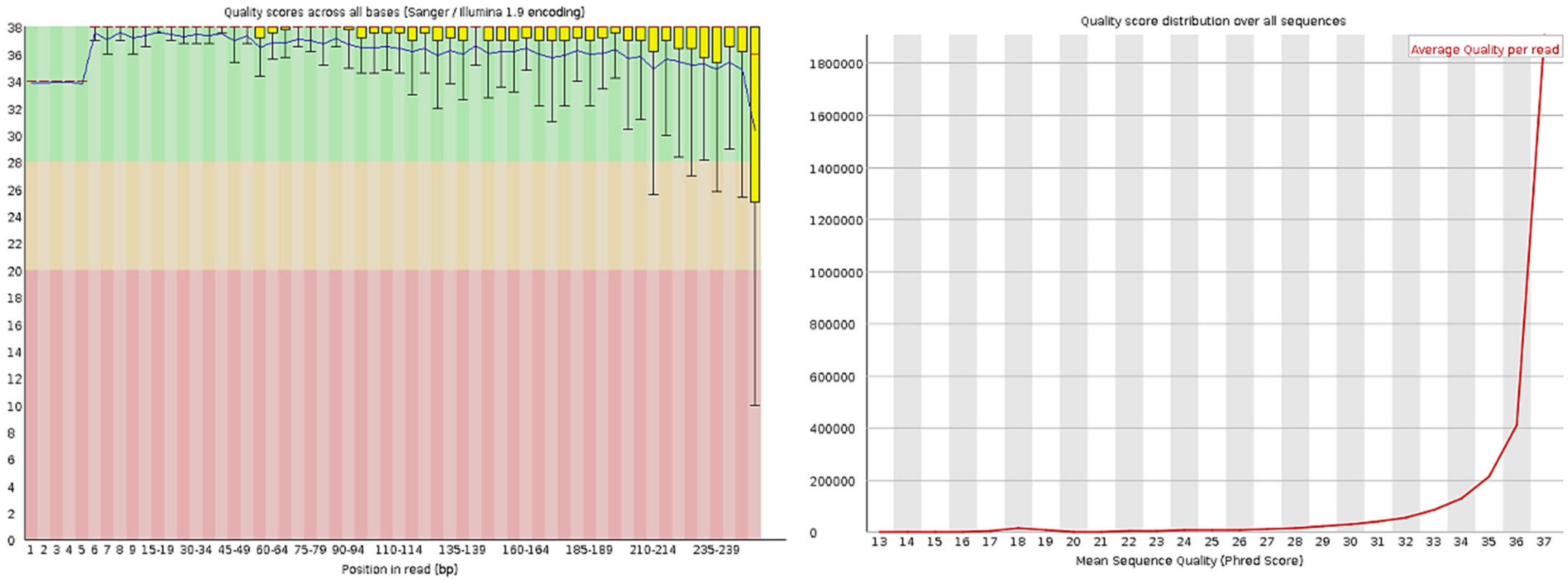

Fig. S2. Bacterial sequence curation and analysis. We sequenced 16Sv4 amplicons generated from DNA samples on a MiSeq. MiSeq-generated Fastq files were quality-filtered and clustered into 97% similarity operational taxonomic units (OTUs) using the mothur software package [http://www.mothur.org]. The per-base raw Q30 (Phred33) scores of the forward sequencing read are summarized (**A**). Sequencing quality for R1 and R2 was determined using FastQC 0.11.5, the per-sequence averaged raw Q30 (Phred33) scores of the forward sequencing read are summarized (**B**).

A

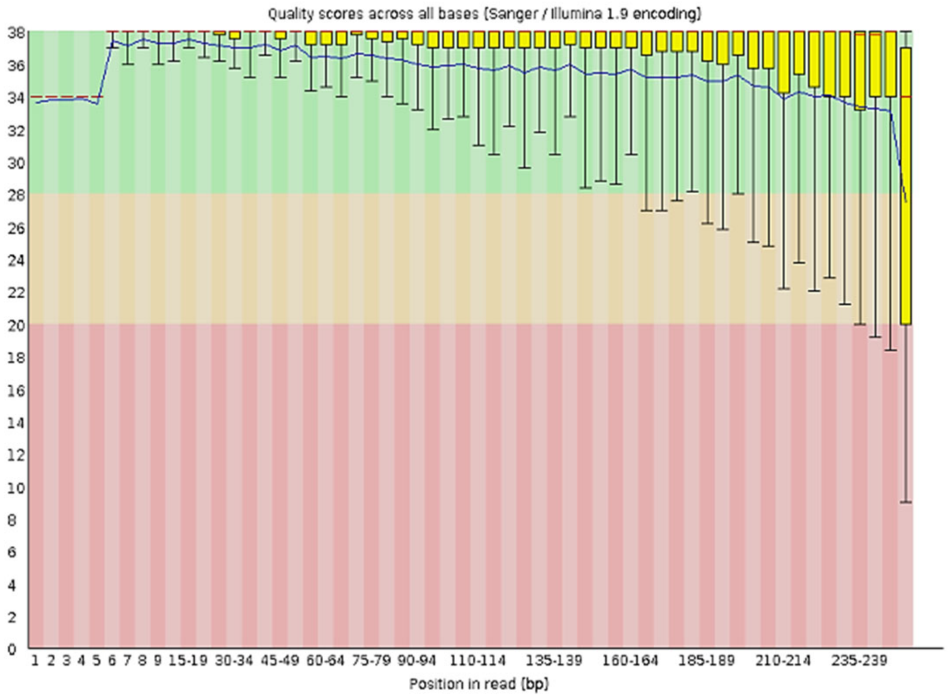

B

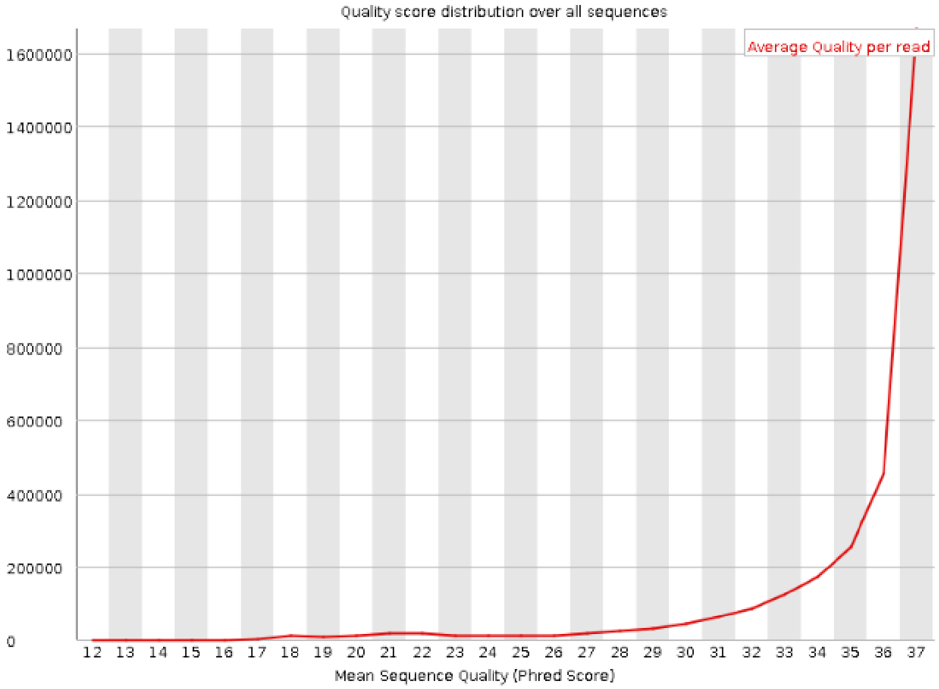

Fig. S3. Fungal sequence curation and analysis. We sequenced ITS2 amplicons generated from DNA samples on a MiSeq. MiSeq-generated Fastq files were quality-filtered and clustered into 97% similarity operational taxonomic units (OTUs) using the mothur software package [http://www.mothur.org]. The per-base raw Q30 (Phred33) scores of the forward sequencing read are summarized (**A**). Sequencing quality for R1 and R2 was determined using FastQC 0.11.5, the per-sequence averaged raw Q30 (Phred33) scores of the forward sequencing read are summarized (**B**).

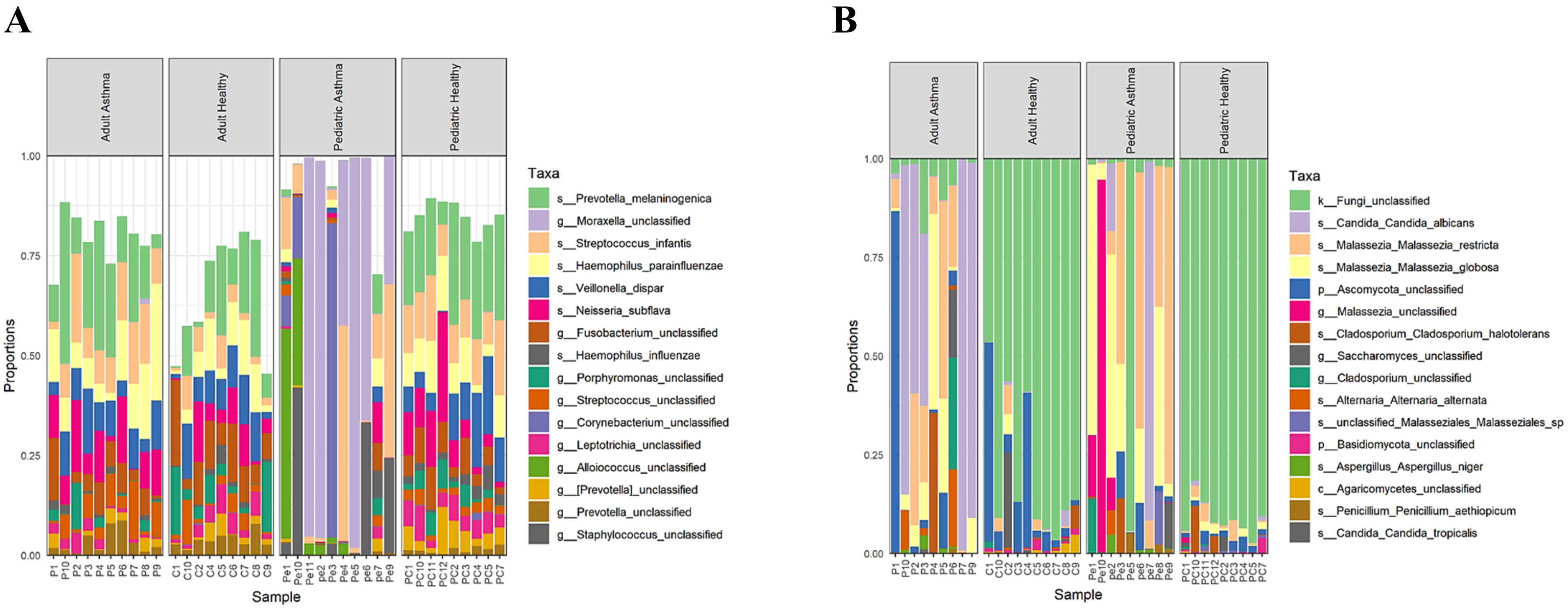

Fig. S4. Aggregated taxonomic composition. High quality reads were classified using Greengenes v. 13_8 as the reference database for bactria and UNITE (v. 7.1) as the reference database for fungi. We aggregated OTUs into each taxonomic rank, the aggregated taxa were visualized at each taxanomic rank using taxanomic bar plots and plotted the relative abundance of the most abundant ones for bacteria (**A**) and Fungi (**B**). The unfilled portion of the bar plots represent lower-abundance taxa.
